# The clinical manifestation and outcome of COVID-19 in patients with a history of ischemic heart disease; a retrospective case-control study

**DOI:** 10.1186/s12872-023-03256-1

**Published:** 2023-05-06

**Authors:** Marzieh Tajmirriahi, Ramin Sami, Marjan Mansourian, Niloufar Khademi, Nastaran-sadat Hosseini, Mehrneagar Dehghan, Forogh Soltaninejad

**Affiliations:** 1 Hypertension Research Center, Isfahan Cardiovascular Research Institute, Isfahan University of Medical Sciences, Isfahan, Iran; 2 Department of Internal Medicine, School of Medicine, Isfahan University of Medical Sciences, Isfahan, Iran; 3 Department of Biology and Biostatistics, School of Public Health, Isfahan University of Medical Sciences, Isfahan, Iran; 4 Faculty of Medicine, Isfahan University of Medical Sciences, Isfahan, Iran; 5 Bamdad Respiratory and Sleep Research Center, Pulmonary ward, Isfahan University of Medical Sciences, Isfahan, Iran; 6 Khorshid Hospital, Ostandari St, Isfahan, Iran

**Keywords:** COVID-19, Ischemic heart disease, Clinical manifestation

## Abstract

**Introduction:**

Coronary artery disease (CAD) is considered an independent risk factor for COVID-19. However, no study has specifically examined the clinical manifestations and outcomes of COVID-19 in patients with ischemic heart disease (IHD).

**Methods:**

In a retrospective case-control study between 20 March 2020 to 20 May 2020, the medical record of 1611 patients with laboratory-confirmed SARS-CoV-2 infection was reviewed. IHD was defined as a history of an abnormal coronary angiography, coronary angioplasty, coronary artery bypass graft (CABG), or chronic stable angina. Demographic data, past medical history, drug history, symptoms, vital signs, laboratory findings, outcome, and death were investigated from medical records.

**Results:**

1518 Patients (882 men (58.1%)) with a mean age of 59.3 ± 15.5 years were included in the study. Patients with IHD (n = 300) were significantly less likely to have fever (OR: 0.170, 95% CI: 0.34–0.81, P < 0.001), and chills (OR: 0.74, 95% CI: 0.45–0.91, P < 0.001). Patients with IHD were 1.57 times more likely to have hypoxia (83.3% vs. 76%, OR: 1.57, 95% CI: 1.13–2.19, P = 0.007). There was no significant difference in terms of WBC, platelets, lymphocytes, LDH, AST, ALT, and CRP between the two groups (P > 0.05). After adjusting for demographic characteristics, comorbidities and vital signs, the risk factors for mortality of these patients were older age (OR: 1.04 and 1.07) and cancer (OR: 1.03, and 1.11) in both groups. In addition, in the patients without IHD, diabetes mellitus (OR: 1.50), CKD (OR: 1.21) and chronic respiratory diseases (OR: 1.48) have increased the odds of mortality. In addition, the use of anticoagulants (OR: 2.77) and calcium channel blockers (OR: 2.00) has increased the odds of mortality in two groups.

**Conclusion:**

In comparison with non-IHD, the symptoms of SARS-CoV-2 infection such as fever, chills and diarrhea were less common among patients with a history of IHD. Also, older age, and comorbidities (including cancer, diabetes mellitus, CKD and chronic obstructive respiratory diseases) have been associated with a higher risk of mortality in patients with IHD. In addition, the use of anticoagulants and calcium channel blockers has increased the chance of death in two groups without and with IHD.

## Introduction

Coronavirus belongs to a family of viruses that are abundant in animals throughout the world. However, few cause disease in humans with symptoms such as pneumonia, fever, breathing difficulty and lung infection [1]. One of these viruses with the current reference name of Acute Respiratory Syndrome Coronavirus 2 (SARS-CoV-2), which has recently caused pathogenesis in humans and quickly caused a pandemic, has been named coronavirus disease 19 [2] by the World Health Organization (WHO) [3, 4]. To date (30 July 2022), about 572 M people have been infected with COVID-19, of which 6.39 M have died. This number is increasing day by day [5].

The clinical manifestation of COVID-19 is non-specific and ranges from asymptomatic to severe conditions and death. [6, 7]. If it is symptomatic, the most frequent symptoms are fever, fatigue, cough, myalgia and complicated dyspnea, whereas less frequent symptoms include hemoptysis, diarrhea, headache and runny nose [1, 8–10]. Comorbidities, i.e., diabetes, hypertension, cirrhosis, cancer, and coronary artery disease (CAD), and aging cause more severe disease and even death [11]. As a common disease, coronary artery disease (CAD) accounts for one-third of the world’s deaths. The most common, life-threatening type of CAD, ischemic heart disease (IHD), is expected to increase from one in 11 cases in 2019 to one in six in 2050 [12]. Therefore, IHD is recognized as a major threat to sustainable development in the 21st century [13–15].

Due to the prevalence of IHD and being a risk factor for severe COVID-19, early diagnosis and proper management of COVID-19 are very important. However, the early detection of these patients requires accurate knowledge of clinical presentations. Despite the high prevalence of IHD and its potential risks associated with COVID-19, no study evaluated the clinical manifestations of SARS-CoV-2 in patients with IHD. Thus, the present study aimed to evaluate the clinical manifestation of COVID-19 in patients with a history of IHD.

## Methods and material

### Study design

This retrospective case-control study was conducted in Khorshid Hospital, the main and first center of COVID-19 in Isfahan province, Iran, from 20 March 2020 to 20 May 2020.

### Patient enrollment

All patients with moderate to severe COVID-19 whose diagnosis was confirmed by real-time reverse transcription polymerase chain reaction (rRT-PCR) test of nasal and pharyngeal swab specimens [10] and outcome of discharge or death prior to 20 May were enrolled in the study. The diagnostic criteria for COVID-19 were made based on the WHO Interim Guideline [16] and severity was defined according to Coronavirus Disease 2019 Treatment Guidelines by the National Institute of Health [17]. Moderate illness was defined as evidence of lower respiratory disease during clinical assessment or imaging, with SpO2 ≥ 94% on room air at sea level. Severe illness was defined as having SpO2 < 94% on room air at sea level, PaO2/FiO2 < 300 mm Hg, a respiratory rate > 30 breaths/min, or lung infiltrates > 50%.

The study consisted of two groups; [1] Case group (IHD) included patients with ischemic heart disease, which was defined as a history of abnormal epicardial coronary artery disease, coronary angioplasty, coronary artery bypass surgery (CABG) and chronic stable angina, and [2] Control group (non-IHD) included patients without any documented coronary artery diseases. Besides, patients with microvascular, whose exercise-related angina or evidence of ischemia in non-invasive tests are associated with no stenoses or mild-to-moderate stenoses (40–60%), revealed by ICA or CTA, that are deemed functionally non-relevant, were included. Abnormal epicardial coronary artery disease was defined as minimal if the narrowing is visually less than 50%, moderate between 50% and 70%, and severe with a diameter reduction of 70% or more [18]. Also, chronic stable angina was defined according to the 2019 ESC Guidelines for the diagnosis and management of chronic coronary syndromes [19]. Initial evaluations identified 1,611 patients with a definitive diagnosis of COVID-19. Medical records of identified patients were evaluated for coronary artery disease documentation and congestive heart failure (CHF). Patients with CHF or acute coronary syndrome were excluded from the study.

### Data collection

The clinical and laboratory data of patients were collected from medical records, including demographics data, underlying disease, drug history, vital signs at admission, clinical symptoms, laboratory findings, comorbid conditions and clinical outcomes.

Comorbidities were recorded based on medical records. The comorbidities are diabetes (DM), chronic kidney disease (CKD), chronic respiratory diseases, hypertension (HTN), end-stage renal disease (ESRD) and malignancies.

Admission vital signs included systolic blood pressure (SBP), diastolic blood pressure (DBP), heart rate (HR), respiratory rate (RR), body temperature [19] and oxygen saturation (SpO2) by pulse oximetry. The abnormal SBP is defined as < 90mmHg (hypotension) or ≥ 140mmHg (hypertension). Also, abnormal DBP, tachycardia, tachypnea, fever (oral temperature) and hypoxemia were defined as ≥ 90mmHg, ≥ 100 beat/min, ≥ 20 beat/min, ≥ 38 °C of and < 93%. Blood pressures were measured by mercury sphygmomanometer (Manufacturer: MDF, 2018, Model: SKU: BP-03,) as Ogedegbe et al. recommendation [20]. Oxygen saturation was assessed by calibrated fingertip pulse oximeter (Manufacturer: Choicemmed, 2019).

In this study, inexpensive and available laboratory tests were considered for the initial evaluation. Complete blood count (CBC) with differential, C-reactive protein (CRP), lactate dehydrogenase (LDH), aspartate aminotransferase [19] and alanine aminotransferase (ALT) were initial laboratory evaluation. In differential CBC, platelets less than 150,000 [21], white blood cells greater than 11,000 or less than 4,000 [22, 23], and lymphocytes less than 1,100 were considered abnormal [24]. Besides, CRP ≥ 10 mg/dl [25], AST > 40 U/L, ALT > 50 U/L and LDH > 250 U/L were considered abnormal [8].

Primary clinical outcomes were defined as hospitalization in the ICU and intubation. Moreover, secondary outcomes were investigated, including length of hospital stay and death.

### Data analysis

Initially, the normal distribution of each variable was investigated by the Kolmogorov-Smirnov test. The chi-square and independent T-test were used to analyze qualitative (categorical) and quantitative data, respectively. The Mann-Whitney test was also used for quantitative data without normal distribution. Categorical and quantitative data were reported in number (percentage) and mean ± standard deviation respectively. The odds ratio and 95% confidence interval were estimated by logistic regression to evaluate the association between symptoms and IHD. In order to estimate the relationship between risk factors related to mortality, multivariable regression was used by adjusting demographic characteristics, comorbidities, used drugs and vital signs. A P-value lower than 0.05 was considered statistically significant. STATA (V.12.0) software performed all the statistical analyses.

## Results

**Fig. 1:**
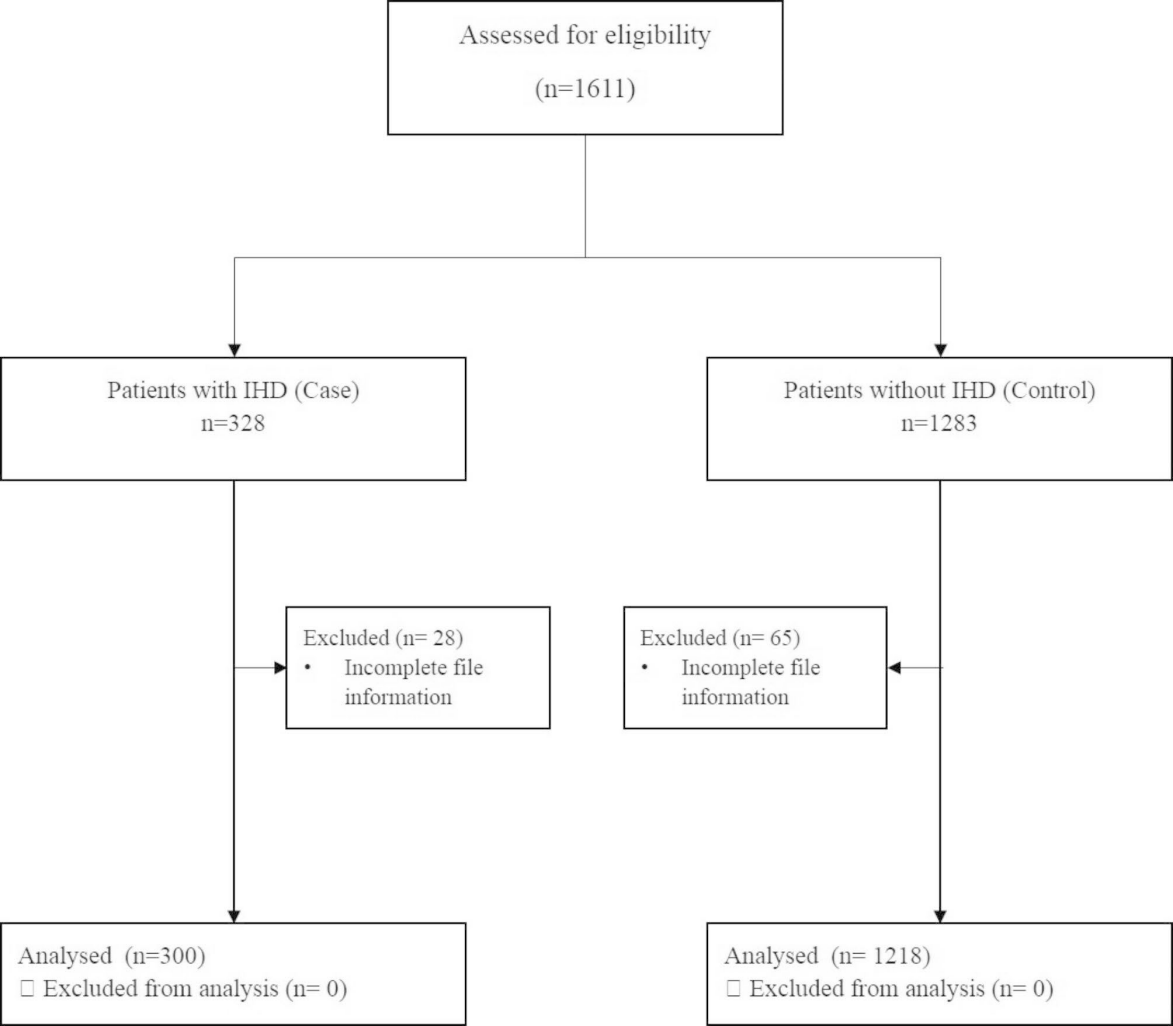
Participant flow diagram

Evaluations of 1611 medical records revealed 328 patients had a history of IHD and 1,283 patients had no history of IHD. Because of missing records, 28 patients from the IHD group and 65 patients from the non-IHD group were excluded from the study. Finally, data were collected from 300 patients in the IHD group and 1218 patients in the non-IHD group (Fig. 1). Of them, 882 (58.1%) patients were men. The mean age was 59.3 ± 15.5 years. Also, there is no significant difference between the two groups regarding gender and smoking (P > 0.05). However, analysis exhibited a significant difference in age between IHD and non-IHD groups, 67.99 ± 11.17 vs. 57.14 ± 15.70 years (P < 0.001). Of the IHD group, 52 and 33 patients had a history of Percutaneous Coronary Intervention and CABG, respectively.

### Comorbidities

Among comorbidities, there was no significant difference between groups regarding the history of chronic respiratory diseases and cancer (P > 0.05). However, the history of HTN, DM, CKD, and ESRD was significantly higher in patients with IHD (P < 0.05) (Table 1).

**Table 1:** Baseline characteristics and comorbidities of patients between two groups with and without HD

Characteristics		Without IHD (N = 1218)	With IHD (N = 300)	P value
**Gender**	**Male**	694(56.9%)	188 (62.7%)	0.074
	**Female**	524(43.1%)	112(73.3%)	
**Age; year**		57.14 ± 15.70	67.99 ± 11.17	**< 0.001**
**Smoking status**		97(7.9%)	33(11%)	0.088
**Comorbidities**				
**DM**		315(25.8%)	147 (49%)	**< 0.001**
**CKD**		48(3.9%)	36(12%)	**< 0.001**
**Chronic respiratory diseases**		109(8.9%)	28(9.4%)	0.861
**HTN**		408(33.5%)	195(65%)	**< 0.001**
**ESRD**		25(2.1%)	18(6%)	**< 0.001**
**Dialysis**		25(2.1%)	17(5.7%)	**0.001**
**Cancer**		40(3.3%)	7(2.4%)	0.611

Data shown mean ± SD or n(%)

DM: Diabetes Mellitus, CKD: Chronic kidney disease, HTN: hypertension, ESRD: End-stage renal disease

### Drug history

Angiotensin-converting enzyme (ACE) inhibitors, beta-adrenergic blockers, calcium channel blockers (CCBs), diuretics, anti-hyperglycemic agents, angiotensin II receptor blockers [19], antilipemic agents, anticoagulant and anticonvulsant were significantly higher in IHD group compared to non-IHD (P < 0.05) (Table 2).

**Table 2:** Frequency distribution of used drugs among two groups with and without HD

Drug categories	Without IHD (N = 1218)	With IHD (N = 300)	P value
**H2 blocker**	784 (64.4%)	221 (73.7%)	0.187
**ACE inhibitor**	21 (1.7%)	11 (3.7%)	**0.036**
**Beta blocker**	118 (9.7%)	103 (34.3%)	**< 0.001**
**calcium blocker**	74 (6.1%)	36 (12%)	**< 0.001**
**Diuretic**	39 (3.2%)	47 (15.7%)	**< 0.001**
**Anti-hyperglycemic**	64 (5.3%)	42 (14%)	**< 0.001**
**LABA**	15 (1.2%)	7 (2.3%)	0.274
**ICS**	15 (1.2%)	5 (1.7%)	0.766
**LABA and ICS**	22 (1.8%)	7 (2.3%)	0.841
**ARB**	203 (16.7%)	109 (36.3%)	**< 0.001**
**Immunosuppressive**	9 (0.7%)	3 (1%)	0.647
**Levothyroxine**	77 (6.3%)	21 (7%)	0.845
**Anti-hyperlipidemic**	155 (12.7%)	126 (42%)	**< 0.001**
**Anticoagulant**	19 (1.6%)	18 (6%)	**0.001**
**ARB and diuretic**	2 (0.2%)	3 (1%)	**0.042**
**SSRI**	41 (3.4%)	8 (2.7%)	0.310
**Anticonvulsant**	49 (4%)	26 (8.7%)	**0.017**
**Antipsychotic**	29 (2.4%)	4 (1.3%)	0.272
**Methylprednisolone**	688 (56.5%)	154 (51.3%)	0.395
**Prednisolone**	55 (4.5%)	19 (6.3%)	0.244

Data shown mean ± SD or n(%)

### Clinical presentations

The five most common symptoms in the IHD group were cough (73.7%), fever (65%), shortness of breath (62.3%), fatigue (59.3%), and chills (50.7%), respectively. On the other hand, the five most common symptoms in the non-IHD group were fever (75.7%), cough (71.8%), shortness of breath (61.6%), chills (60.1%), and weakness and fatigue (59.4%), respectively. Analyses showed patients with IHD were significantly less likely to present symptoms, including fever (OR: 0.170, 95% CI: 0.34–0.81, P < 0.001) and chills (OR: 0.74, 95% CI: 0.45–0.91, P < 0.001). The other symptoms had no significant difference between the two groups (P > 0.05) (Table 3).

**Table 3:** Comparison of the frequency distribution of vital signs, clinical presentations, and laboratory parameters between two groups with and without HD

Variable	Without IHD (N = 1218)	With IHD (N = 300)	OR (95% CI)^*^	P value
**Fever**	922(75.7%)	195(65%)	0.170(0.34–0.81)	**< 0.001**
**Chill**	732(60.1%)	152(50.7%)	0.74(0.45–0.91)	**< 0.001**
**Cough**	874(71.8%)	221(73.7%)	1.63(0.83–1.65)	0.198
**Sore throat**	205(16.8%)	46(15.3%)	0.20(0.11-1.00)	0.827
**Loss of smell**	162(13.3%)	33(11%)	0.78(0.52–1.45)	0.368
**Sneeze**	85(7%)	15(5%)	0.65(0.43–1.03)	0.836
**Runny nose**	124(10.2%)	21(7%)	0.70(0.30–1.07)	0.714
**Body pain**	689(56.6%)	151(50.3%)	0.64(0.45–1.10)	0.101
**Nausea**	439(36%)	95(31.7%)	0.84(0.65–1.13)	0.883
**Vomiting**	285(23.4%)	61(20.3%)	0.80(0.57–1.01)	0.211
**Diarrhea**	332(27.3%)	59(19.7%)	0.71(0.40–1.01)	0.510
**Short of breath**	750(61.6%)	187(62.3%)	1.03(0.81–1.61)	0.832
**Decreased appetite**	535(43.9%)	143(47.7%)	1.28(0.89–1.85)	0.181
**Abdominal Pain**	190(15.6%)	46(15.3%)	1.01(0.65–1.35)	0.989
**Headache**	413(33.9%)	84(28%)	0.68(0.87–1.11)	0.155
**Chest Pain**	299(24.5%)	64(21.3%)	0.71(0.56–1.06)	0.687
**Fatigue**	724(59.4%)	178(59.3%)	1.00 (0.68–1.36)	0.991
**Weight Loss**	215(17.7%)	45(15%)	0.83(0.64–1.12)	0.830
**Nasal Congestion**	48(3.9%)	8(2.7%)	0.65(0.42–1.24)	0.204
**Symptom duration**	8.23 ± 6.12	7.74 ± 5.67	0.70(0.45–1.12)	0.512
**SBP; mmHg**	132 ± 22.03	131.58 ± 57.46	1.01(0.68–1.18)	0.800
**DBP; mmHg**	80.11 ± 35.15	79.1 ± 13.97	1.02(0.60–1.26)	0.555
** h on admission; beat/min**	93 ± 15.72	90.02 ± 17.33	0.75(0.46–0.96)	**0.003**
**RR on admission; beat/min**	22.42 ± 6.34	22.96 ± 6.04	1.00(0.55–1.11)	0.898
**Temperature on admission; °C**	37.52 ± 0.99	37.42 ± 2.21	1.01(0.80–1.30)	0.500
**SaO2**	87.85 ± 27.46	85.1 ± 10.34	0.77(0.09–0.86)	**0.026**
**Laboratory parameters**				
**PLT**(×10^3^/**µL**)	192.84 ± 76.34	191.61 ± 77.86	0.99(0.99-1.00)	0.511
**WBC**(/**µL**)	6225.22 ± 8618.48	6233.31 ± 3301.1	1.00(1.00-1.01)	0.596
**Lymph**(/**µL**)	1199.2 ± 4620.94	1075.4 ± 686.43	0.99(0.96–1.01)	0.385
**LDH (U/L)**	690.43 ± 586.59	675.59 ± 457	0.99(0.98–1.01)	0.570
**AST(U/L)**	46.85 ± 39.07	45.53 ± 32.84	1.01(1.00-1.02)	0.083
**ALT (U/L)**	37.24 ± 40.2	32.7 ± 27.62	0.98(0.97–1.01)	0.128
**CRP(mg/l) (0–10 mg/l)**	58.21 ± 53.42	56.45 ± 53.45	0.99(0.98–1.01)	0.268

Data shown mean ± SD or N(%)

*: adjusted for age, sex, drugs, comorbidities

OR(95% CI): Odd ratio(95% Confidence interval) SBP: Systolic blood pressure; DBP: Diastolic blood pressure; HR: Heart rate; RR: Respiratory rate; Plt: Platelet; WBC: White blood cell; Lymph: Lymphocyte; LDH: lactate dehydrogenase; AST: Aspartate transaminase; ALT: Alanine transaminase; CRP: C-reactive protein

### Vital signs

At the time of admission, body temperature (T) (37.42 ± 2.21 vs. 37.52 ± 0.99 °C, P = 0.500), DBP (79.1 ± 13.97 vs. 80.11 ± 35.15 mmHg, P = 0.555), SBP (131.58 ± 57.46 vs. 132 ± 22.03 mmHg, P = 0.800) and tachycardia (27.3% vs. 33%, P = 0.059) were not statistically significantly different between IHD and non-IHD groups. However, the mean HR (90.02 ± 17.33 vs. 93 ± 15.72 beat/min, P = 0.003) and SaO2 (85.1 ± 10.34 vs. 87.85 ± 27.46%, P = 0.026) were significantly lower in IHD group.

Laboratory findings.

A quantitative comparison of laboratory data showed that white blood cell counts, platelets, lymphocytes, lactate dehydrogenase (LDH), aspartate transaminase [19], alanine transaminase (ALT) and C-reactive protein (CRP) were not significantly different between the two groups (Table 3).

### Clinical outcomes

Length of hospitalization was significantly higher in the IHD group (8.4 ± 5.57 days vs.7.5 ± 5.5 days, P = 0.042). However, the ICU admission, ICU length of stay, the need to mechanical ventilation, duration of mechanical ventilation, death and time from admission to death were not significantly different between the IHD and non-IHD groups (P > 0.05) (Table 4).

**Table 4:** Comparison of patient outcome in two groups with and without HD

Outcomes	Without IHD (N = 1218)	With IHD (N = 300)	OR (95% CI)^*^	P value
**ICU admission**	181 (14.9%)	47 (15.7%)	0.789(0.52–1.20)	0.265
**ICU length of stay; day**	8.96 ± 7.21	9.45 ± 6.16	1.04(0.76–1.40)	0.818
**Mechanical ventilation**	61 (5%)	16 (5.3%)	0.94(0.532–1.64)	0.771
**Duration of mechanical ventilation; day**	8.81 ± 8.05	8.44 ± 6.82	1.05(0.78–1.43)	0.051
**Death**	92 (7.6%)	30 (10%)	1.03(0.79–1.10)	0.349
**Time to death; day**	13.1 ± 9.41	11.32 ± 7.78	1.01(0.95–1.07)	0.686
**Length of hospitalization; day**	7.5 ± 5.5	8.4 ± 5.7	1.12(1.01–1.85)	0.042

Data shown mean ± SD or N(%)

*: adjusted for age, sex, drugs, comorbidities, vital signs and respiratory

Finally, the results of the multivariate regression analysis of mortality risk factors in each of the two groups with and without IHD showed that in patients without IHD, age, length of hospitalization, use of anti-coagulants, comorbidities (including chronic respiratory diseases, diabetes, cancer and CKD) significantly increase the odds of mortality (P < 0.05). However, increasing DBP and SaO2 decreased the odds of mortality (P < 0.05). In the group of patients with IHD, older age, use of calcium channel blockers, increased HR, and RR and having cancer can increase the odds of mortality (P < 0.05) (Table 5).

**Table 5:** Risk factors of mortality in each of the two groups with and without HD

Risk factors of mortality		OR (95% CI)^*^	P value
**Without IHD**	**Age; year**	1.04(1.01–1.06)	0.005
	**Length of hospitalization; day**	1.12(1.06–1.14)	< 0.001
	**Anti-coagulation**	2.77(1.45–5.30)	0.002
	**DBP**	0.97(0.94–0.99)	0.023
	**SaO2**	0.89(0.84–0.92)	< 0.001
	**Chronic respiratory diseases**	1.48(0.25–0.91)	0.025
	**Diabetes**	1.50(1.29–1.87)	0.012
	**Cancer**	1.03(1.01–1.81)	0.017
	**CKD**	1.21(1.10–1.47)	< 0.001
**With IHD**	**Age; year**	1.07(1.01–1.16)	0.047
	**Calcium channel blockers**	2.00(1.54–3.14)	0.023
	**HR**	1.04(0.97–1.12)	0.024
	**RR**	1.76(1.52–2.11)	0.041
	**Cancer**	1.11(1.02–1.68)	0.018

*: Multivariable regression adjusted for age, sex, used drugs, comorbidities, and vital signs and respiratory

OR(95% CI): Odd ratio(95% Confidence interval), DBP: Diastolic blood pressure; HR: Heart rate; RR: Respiratory rate; CKD: Chronic kidney disease

## Discussion

The present study on “comparing the clinical and laboratory characteristics and outcomes of patients with IHD and patients without IHD” reported that the five most common symptoms in patients with IHD were cough, fever, shortness of breath, fatigue and chills. On the other hand, the five most common symptoms in patients without IHD were fever, cough, shortness of breath, chills and fatigue. Only three symptoms, fever, chills and diarrhea, were significantly less frequent in IHD patients. The mean heart rate and systolic blood pressure were significantly lower in IHD patients. Also, hypoxemia was significantly higher in patients with IHD. Among clinical outcomes, only the hospital stay of patients with IHD was significantly longer. Also, it was shown that older age, use of calcium channel blockers, increased HR, and RR and positive history of cancer were associated with increased mortality in patients with IHD.

In the previous studies, the early stages of the SARS-CoV-2 infection are characterized by mild constitutional symptoms and upper respiratory tract infection. The most common symptoms in the early stages of the disease, the viral phase, include fever, cough, sore throat, shortness of breath, malaise, fatigue and headache [26–28]. Subsequently, nasal congestion, rhinorrhea and sneezing may occur [1, 8, 26, 29, 30]. The inflammatory phase, which usually begins in the second week of the disease, manifests with symptoms such as dyspnea, tachypnea, hypoxemia, diarrhea, and abdominal pain [10, 26, 27, 31]. These symptoms are due to RNAemia and cytokine release [2, 6, 21, 26, 29]. The presence of viral phase and inflammatory phase symptoms in our patients may be justified by not classifying patients at different times from the onset of symptoms to hospitalization.

A case-control study on 859 patients, 113 patients with heart disease (HD) and 746 without heart disease, by Gonzalo Cabezón Villalba et al. reported that the mean age of patients with heart disease compared to the control group was significantly higher, 75.6 vs. 67 (P < 0.001) [32]. Besides, the history of DM, CKD and HTN in the HD group was significantly more than the non-HD group (38.1 vs. 16.5%, 14.2 vs. 5.8%, 76.1 vs. 45.4%, P < 0.001). Our findings in patients with IHD also supported their findings regarding the mean age and comorbidities.

The study by Gonzalo Cabezón Villalba showed that cough is the only symptom that occurs significantly lesser in patients with heart disease. Regarding fever and diarrhea, they did not find any significant difference [32]. however, our study revealed that patients with a history of IHD reported less fever, chills and diarrhea than the control group. It seems that these symptoms could not help diagnose because patients with IHD usually receive drugs such as acetylsalicylic acid (ASA) and calcium channel blockers (CCBs) like amlodipine verapamil and diltiazem as secondary prevention. ASA has both anti-inflammatory (high doses) and antithrombotic effects (low doses) [33]. However, ASA is prescribed in low doses, 80 mg/day, in case of secondary prevention in patients with IHD. Thus, it could be suggested that ASA is not responsible for reducing these symptoms in these patients. In addition, a side effect of calcium channel blockers is constipation resulting from colonic motor activity inhibition [34, 35]. Therefore, patients consuming calcium channel blockers may report diarrhea lesser than healthy people.

The study on patients with heart disease reported no significant difference in the complaint of dyspnea between groups [32]. Our study is in line with their study. The onset of dyspnea and tachypnea were not statistically significantly different between patients with and without IHD. Although patients with IHD had less oxygen saturation than the control group, the complaint of dyspnea had no significant difference. Nevertheless, we do not have a proper explanation for this finding. The accuracy of this finding needs further study in the future.

The prevalence of CAD in patients with COVID-19 was variable between 2.5 and 40% in previous studies [8, 21, 28, 36]. In the present study, 20% of the patients had a history of IHD. Some studies have cited the association between cardiovascular disease (CVD) and severe COVID-19 as a secondary outcome of their study. The death rates of patients with CAD were significantly higher compared with other patients in the study of Zhou et al. (24% vs. 1%, p < 0.0001) and Wu et al. (10.5% vs. 2.3%, P < 0.001) [37, 38]. Mehra et al. cited coronary artery disease (10.2%, vs. 5.2%, OR: 2.70, 95% CI: 2.08–3.51), heart failure (15.3%, vs. 5.6%, OR: 2.48, 95% CI: 1.62–3.79), and cardiac arrhythmia (11.5%, vs. 5.6%, OR: 1.95; 95% CI: 1.33–2.86) as independent factors associated with an increased risk of hospital death in patients with COVID-19 [39]. Gonzalo Cabezón Villalba et al. reported that the in-hospital mortality in patients with heart disease was higher than the control group (35.4% vs. 18.2%, p < 0.001). However, in the present study, IHD did not increase adverse outcomes and mortality rates in patients with COVID-19. This discrepancy is probably due to our definition of IHD patients. Though previous studies considered all types of heart disease as CAD, the definition of IHD in the present study was only considered as a positive history of abnormal coronary angiography, coronary angioplasty, CABG and chronic stable angina. In addition, by adjustment of other risk factors, e.g., comorbidities and age, only the net effect of IHD was assessed.

In a comparison of our results with studies on a healthy population, a systematic review and meta-analysis of 42 studies and 423,117 patients showed that acute kidney injury, COPD, diabetes, hypertension, CVD, cancer, increased D-dimer, male gender, older age, current smoker, and obesity are associated with a higher mortality rate [40]. In our study, some of these factors, including cancer, COPD, older age, diabetes and hypertension, have been identified as risk factors in the non-IHD group.

We had some limitations. First, the unavailability of echocardiography results and the nature of the study (retrospective) made us diagnose CHF only based on prior medical records and/or symptoms and signs. Also, we excluded patients with CHF because the cause of heart failure, i.e., ischemic or structural, was not known. Second, since Our hospital was the center for COVID-19 patients in the region only during the mentioned time (20 March 2020 to 20 May 2020), it could not be possible to include further subvariants of SARS-CoV-2 in the study and this study just assessed the very first peak of COVID-19.

## Conclusion

According to the results of the current study, age, comorbidities (including diabetes, CKD, HTN, ESRD, and dialysis), fever, and length of hospitalization in the IHD group were significantly more than in the group without IHD. While chill symptoms and high HR in the group without IHD were significantly more than the IHD group. In the evaluation of the risk factors related to the mortality of these patients, it was found that patients with older age and cancer in both groups were associated with a higher risk of mortality. In addition, in the group of patients without IHD, diabetes, CKD and chronic respiratory diseases have increased the odds of mortality. In addition, the use of anticoagulants and calcium channel blockers has increased the odds of mortality in two groups without and with IHD, respectively.

## Data Availability

• The datasets used and/or analysed during the current study available from the corresponding author on reasonable request.

## List of abbreviations

IHD: Ischemic Heart Disease
CAD: Coronary Artery Disease
CABG: Coronary Artery Bypass Graft
DM: Diabetes Mellitus
CKD: Chronic Kidney Disease
HTN: Hypertension
ESRD: End Stage Renal Disease
